# Genetic Diversity of Oilseed Rape Fields and Feral Populations in the Context of Coexistence with GM Crops

**DOI:** 10.1371/journal.pone.0158403

**Published:** 2016-06-30

**Authors:** Diane Bailleul, Sébastien Ollier, Jane Lecomte

**Affiliations:** 1 IFREMER, UMR MARBEC, Station de Sète, Avenue Jean Monnet, CS 30171, 34203 Sète Cedex, Sète, France; 2 Ecologie Systématique Evolution, Univ. Paris-Sud, CNRS, AgroParisTech, Université Paris-Saclay, 91400, Orsay, France; Chungnam National University, REPUBLIC OF KOREA

## Abstract

Despite growing concern about transgenes escaping from fields, few studies have analysed the genetic diversity of crops in an agroecosystem over several years. Accurate information about the dynamics and relationship of the genetic diversity of crops in an agroecosystem is essential for risk assessment and policies concerning the containment of genetically modified crops and their coexistence with crops grown by conventional practices. Here, we analysed the genetic diversity of oilseed rape plants from fields and feral populations over 4 years in an agricultural landscape of 41 km^2^. We used exact compatibility and maximum likelihood assignment methods to assign these plants to cultivars. Even pure lines and hybrid cultivar seed lots contained several genotypes. The cultivar diversity in fields reflected the conventional view of agroecosystems quite well: that is, there was a succession of cultivars, some grown for longer than others because of their good performance, some used for one year and then abandoned, and others gradually adopted. Three types of field emerged: fields sown with a single cultivar, fields sown with two cultivars, and unassigned fields (too many cultivars or unassigned plants to reliably assign the field). Field plant diversity was higher than expected, indicating the persistence of cultivars that were grown for only one year. The cultivar composition of feral populations was similar to that of field plants, with an increasing number of cultivars each year. By using genetic tools, we found a link between the cultivars of field plants in a particular year and the cultivars of feral population plants in the following year. Feral populations on road verges were more diverse than those on path verges. All of these findings are discussed in terms of their consequences in the context of coexistence with genetically modified crops.

## Introduction

The introduction of genetically modified (GM) plants in agroecosystems formed the starting point of the study of gene flows in human-shaped environments [1]. The ability of GM plants to introgress with non-GM plants is an issue for coexistence between GM and non-GM crops in agroecosystems and raises concerns about GM contaminations through the link between cultivar seeds and harvests [2].

Oilseed rape (OSR) is a model plant for studying gene flow at the landscape scale. OSR is an economically important crop in North America and Europe. GM OSR is mainly cultivated in North America, but its cultivation in Europe is a matter of controversy. OSR exhibits traits that suggest that it was recently domesticated [3]: partial autogamy, seeds with a strong capacity for dehiscence before harvest and significant secondary dormancy.

OSR pod shattering is a common phenomenon, which causes a mean loss during harvest of 8000 seeds per m^2^ [4]. On average, this represents 9 to 56 times the number of seeds sown [5]. OSR seeds have the ability to establish long-lived seed banks in the soil via secondary dormancy. This ability to become dormant differs depending on the cultivar [6–8] and the cultivation conditions, such as soil tillage [6,9]. Studies have shown that seeds survived for at least 5 years in Canada [9] and 17 years in Europe [2,10,11]. Alongside these seed banks, OSR volunteers are common in fields [9]. Another consequence of pod shattering and seed banks is the development of populations of crops outside fields, which are called feral populations. OSR feral populations are common along roadsides [12–17] and railways [18–20] and thus could originate from feral seed banks [17], from the harvesting of adjacent fields [17], from truck spillage [12,21], from grain trailers [22] and from vehicular transport [23–25].

As GM plants have the ability to introgress with non-GM relatives, GM genes are frequently used as markers of gene flow. Indeed, cross-pollination between OSR plants can be clearly characterized by identifying GM genes [26,27]. GM pollen flows via cross-pollination between OSR plants were detected across distances up to 800 m [28], 1100 m [29] and 3000 m [30]. The existence of GM volunteers [2,31,32] and GM feral plants [14,15,19,33] could be either a consequence or a cause of GM elusion from human management.

Another consequence of the ability of OSR to establish feral and volunteer populations and achieve cross-pollination is the appearance of plants with multiple GM traits [15,34–36] that do not exist in cultivars. Certified seed lots have also been shown to be contaminated by GM genes [28,37,38]. Recent studies have shown that, after growing GM cultivars in a field, it is impossible to meet the European threshold for ‘no GM’ labelling for conventional cultivars in subsequent years [2,39].

Previous studies have surveyed OSR feral populations at different scales of time or space, and with multiple cultivars. Pascher et al. [40] examined eight individuals from each of eight feral populations in the same year with regard to 19 cultivars and nine SSR (Single Sequence Repeat) in order to distinguish them. They observed high genetic differentiation between feral populations and cultivars and deduced that feral populations maintained themselves over time by self-recruitment and hybridisation with cultivars in fields. Bond et al. [41] compared a six-year-old feral population with 13 cultivars. A small proportion of the feral plants were similar to the cultivars, indicating that they originated via direct seed spillage. However, as most of the feral plants were genetically different from the cultivars, harvest seed spillage did not appear to have been the main origin of the feral population. Finally, Elling et al. [42] examined 161 individuals from 18 feral populations from 2004 to 2007, and seven cultivars. By a maximum likelihood assignment method, they identified four different sources of feral populations and three intercultivar hybrids. However, studies on local spatial scales and/or over a short period are not appropriate to draw definitive conclusions on the origins and persistence of feral populations. Studies on a large spatial scale are necessary to take into account the entire diversity of agricultural practices, such as seed spillage by grain trucks at harvest [22]. Moreover, information about the genetic composition of OSR cultivars is not sufficient to obtain an accurate understanding of the genetic composition of fields. Indeed, several factors could lead to the presence of multiple cultivars in the same field: volunteers, seed lot contamination (estimated to occur at a level of 8.7% of seeds) [43], pollination from other sources (5% of seeds) [43] and agricultural practices (farm-saved seeds and the sowing of several cultivars in a single field).

Here we present a 4-year survey of OSR field and feral plants in an agroecosystem. Population genetics tools were used to assign each sampled plant to a cultivar and to estimate the origin of the feral populations. Our investigation focused particularly on the number of cultivars in each field, the temporal evolution of genetic diversity in feral populations, and the link between the diversity of cultivars in fields and that in feral populations.

## Materials and Methods

No specific permission was required for this study as french roads are public areas. Road verges are not privately-owned or protected. The DDE (Direction Départementale de l'Equipement, in charge of road verges management), town mayors and farmers were informed about the study before the experiment. The field studies did not involve endangered or protected species.

### Study area

**Fig 1:**
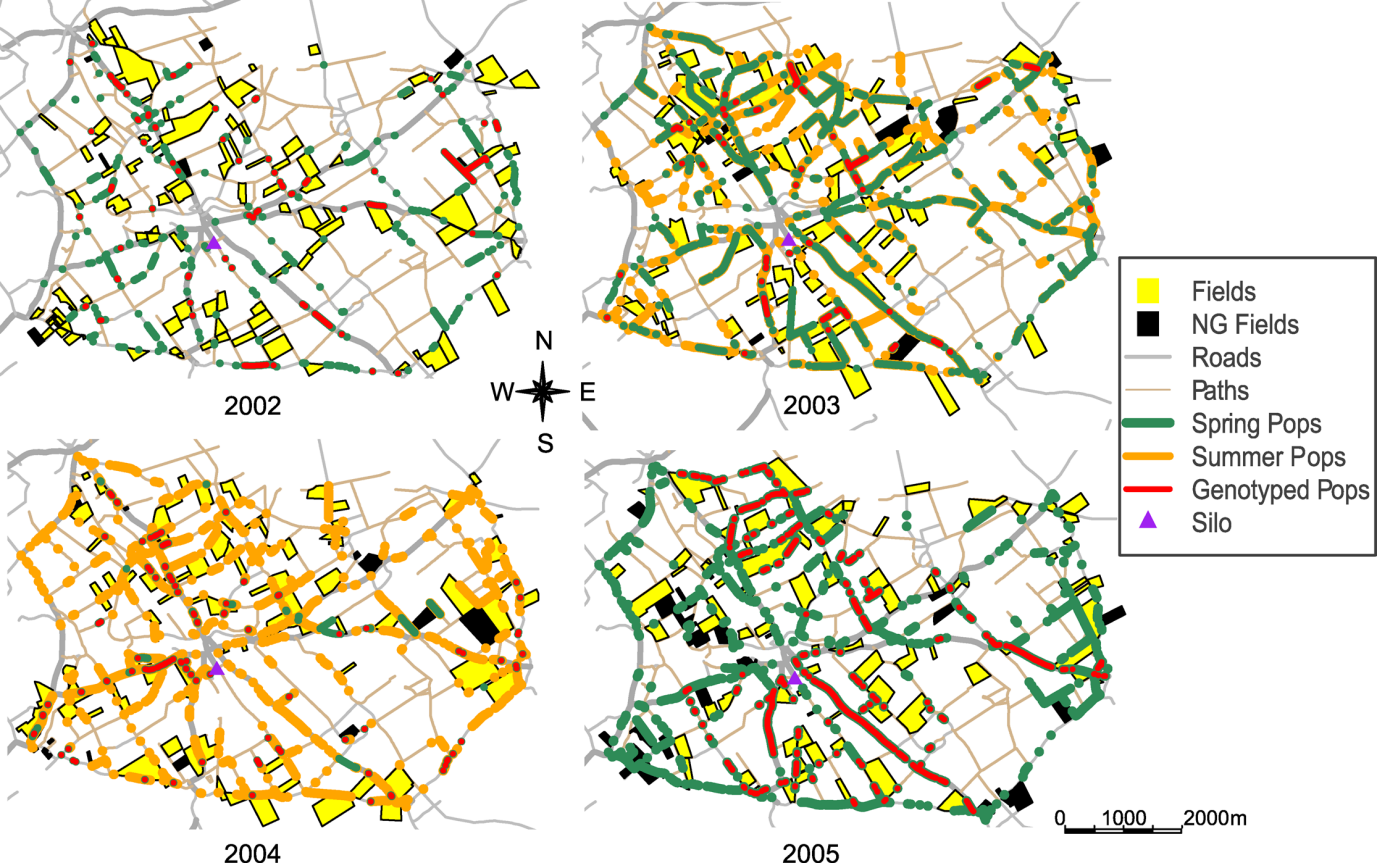
Map of the Selommes area, France, with the positions of fields and populations studied between 2002 and 2005. *NG*: populations not genotyped. *Genotyped pops*: populations collected and genotyped. In 2002 and 2005, only spring populations (i.e. *Spring pops*) were mapped and collected. In 2004, summer populations (*Summer pops*) mainly overlapped with spring populations.

The study area (Fig 1) is a typical open-field agricultural landscape of 41 km^2^ centred on the village of Selommes, Loir-et-Cher, France (47°45'24''N, 1°11'34''E), which contains a grain silo where most local farmers take their harvested grain.

From 2002 to 2005, a census was carried out twice a year along the 110 km of roads within the study area: the first during OSR flowering (mid-April) and the second before harvesting (end of June to early July). The survey was performed from a car driving at a maximum speed of 15 km.h^-1^. Field and feral populations were recorded and mapped. Roads were categorised as paths (42% of roads) and paved roads (58%). In each year, fields in which OSR was cultivated represented 11% to 14% of the total area, and numbered 100 on average [17]. Feral plants were not recorded individually, but all plants separated by less than 10 m were considered to belong to the same population. Feral populations were found only on road verges and were present on 10%–14% of their length across years [17].

### Data collection

OSR plants were sampled at the edges of roads. From 2002 to 2003, seeds were taken from mature pods from each sampled plant. Ten plants were sampled per field. A maximum of 10 plants were sampled per feral population. In July 2004, leaves and seeds were sampled from each plant. In April 2005, only leaves were sampled.

From 2003 to 2005, we analysed 3010 field plants belonging to 269 OSR fields (among the 330 fields in the area; Table 1). Genetic data from the fields in 2002 was reported previously [44]. From 2002 to 2005, we analysed 2300 plants from 497 feral populations (among 2904 total populations: 1903 populations in April and 1001 populations in June).

**Table 1:** Sampling from 2003 to 2005 (fields) and 2002 to 2005 (feral populations).

	Verges/populations	Plants	Seeds	Plants not assigned
2003 fields (S)	94	987	2026	161 (16.3%)
2004 fields (L)	96	687	-	216 (31.4%)
2004 fields (S)	79	412	832	76 (18.4%)
2005 fields (L)	96	924	-	265 (28.7%)
2002 populations (S)	95	318	2543	93 (29.2%)
2003 populations (S)	62	176	1363	69 (39.2%)
2004 populations (L)	63	178	-	61 (34.3%)
2004 populations (S)	49	188	1436	63 (33.5%)
2005 populations (L)	247	1440	-	493 (34.2%)

Number of plants sampled using leaves (L) and seeds (S) from different fields and feral populations. For example, in 2004, for fields, 687 leaves were sampled from 687 plants (31.4% were not assigned to cultivars) and 832 seeds were sampled from 412 plants (18.4% were not assigned).

A list of 58 cultivars that were potentially sown in the area of Selommes from 2002 to 2005 was established from a long-term survey of farmers and from Terres Inovia technical center [4] data. We obtained seeds from seed companies from these cultivars: 45 pure-line cultivars (homogeneous homozygous), 11 hybrid cultivars (homogeneous heterozygous) and 2 cultivar associations (heterogeneous genotypes).

### Molecular markers

#### Laboratory work

We selected seven SSR markers (Table 2) that exhibited the highest polymorphism and allowed the discrimination of our cultivars: Ra2E11, Na12D08, Na10H03, Ol12F02, Ol11B05, Na14H11 and Ra2A05. Primers for Ol11B05, Na14H11 and Ra2A05 amplified two loci each, one of which was monomorphic and so was not considered. Primers for Ol12F02 amplified two polymorphic loci. Thus, in total, eight usable polymorphic and independent loci were amplified from these seven SSR markers. The polymorphism rate varied from two to seven alleles by locus. No null alleles were detected.

**Table 2:** Alleles obtained using primers to amplify loci in samples obtained from 2003 to 2005.

Locus	Primers	Alleles
**1**	Ra2E11	189 206 210 218 220
**2**	Na12D08	94 95 100 114 127 132 136
**3**	Na10H03	128 132
**4**	Ol12F02-A	132 138
**5**	Ol12F02-B	153 162 164 166 192
**6**	Ol11B05	130 138
**7**	Na14H11	123 126 128
**8**	Ra2A05	99 102 104

Molecular laboratory work was conducted using the genotyping platform of Clermont-Ferrand (INRA, France). DNA was extracted by a modified version of a previously described method [45]. The M13-tailed primer was used to fluorescently label the polymerase chain reaction (PCR) products [46]. The seven primer pairs were used in duplexes.

#### Genetic data

During spring, we sampled and genotyped one leaf per collected plant, whether it was from fields or feral populations. During harvest, leaves were generally in bad conditions so we either sampled and genotyped two seeds per field plant and eight seeds per feral plant. We took extra seeds for feral plants because we supposed cultivar assignment would be more troublesome with plants of unknown origins.

We genotyped a total of 4469 samples from fields: 1611 leaves and 2858 seeds. We genotyped 6960 samples from feral populations: 1618 leaves and 5342 seeds.

As for the cultivars, we genotyped from 10 to 68 seeds per cultivar (mean = 25, SD = 12.64, SE = 1.66) considering the cultivar type (pure line, hybrid or association). We genotyped a total of 1469 cultivar seeds.

### Field and feral plant assignment

To assign a cultivar to each plant sampled, we used assignment methods based on genotype data: exact compatibility assignment and maximum likelihood assignment. Leaves were assigned by the direct method of exact compatibility assignment: if the plant genotype is compatible for all of the loci with one of the genotypes of a given cultivar, the plant is assigned to this cultivar. A maximum likelihood assignment method was developed to assign a cultivar to each of the plants sampled using seeds (S2 Supporting Information) [43]. We did not allow cross-recombination among cultivars, only within cultivars; we also did not consider intercultivar hybrids. If the findings were ambiguous, the plant was assigned to the most consensual cultivar with the highest likelihood. A field was assigned to a particular cultivar if at least six of the ten sampled plants belonged to the same cultivar (S1 Supporting Information).

### Exclusion probability

Genotypic frequencies within cultivars were used to compute the likelihood of plant assignation and to evaluate the exclusion power of the eight loci. For this purpose, we computed two exclusion probabilities: the classical exclusion probability from Jamieson and Taylor [47], and a derived exclusion probability taking into account the cultivar frequencies of plants in field and feral populations in our data (S3 Supporting Information), adapting the exclusion probability from Devaux et al. [43].

### Cultivar diversity of interfield and interferal populations

We used a double principal coordinate analysis (DPCoA: Double Principal Coordinate Analysis) [48] method for the assignment of data transformed into cultivar richness to describe the presence of similarity between cultivars in fields (interfield) and in feral populations (interferal) for each year.

Exclusion probabilities were computed with Mathematica [33]. Data were implemented and mapped with R software [44].

## Results

### Field and feral population genotypes

We did not find any significant difference between the proportions of genotypes for field and feral populations that had more than six missing alleles (paired samples Wilcoxon test). We only considered genotypes with a maximum of six missing alleles.

We obtained 3893 genotypes for field populations and 6080 genotypes for feral populations. We obtained from 5 to 51 full genotypes per cultivar (mean = 20, SD = 8.89, SE = 1.17). A mean of 5.5 unique genotypes per cultivar was found: 3.9 genotypes for pure line cultivars, 9.7 for hybrid cultivars and 20 for cultivar associations.

We assigned the cultivar genotypes to themselves, and when the likelihood probabilities of assignment for some pairs of cultivars were similar, an *a posteriori* grouping was made. This was the case for cultivars Cadillac and Canary (CDL.CNY), Capitol and Carolus (CPL.CRS), and Gaspard and Samouraï (GPD.SMI).

### Field and feral plant assignment

#### OSR fields

**Fig 2:**
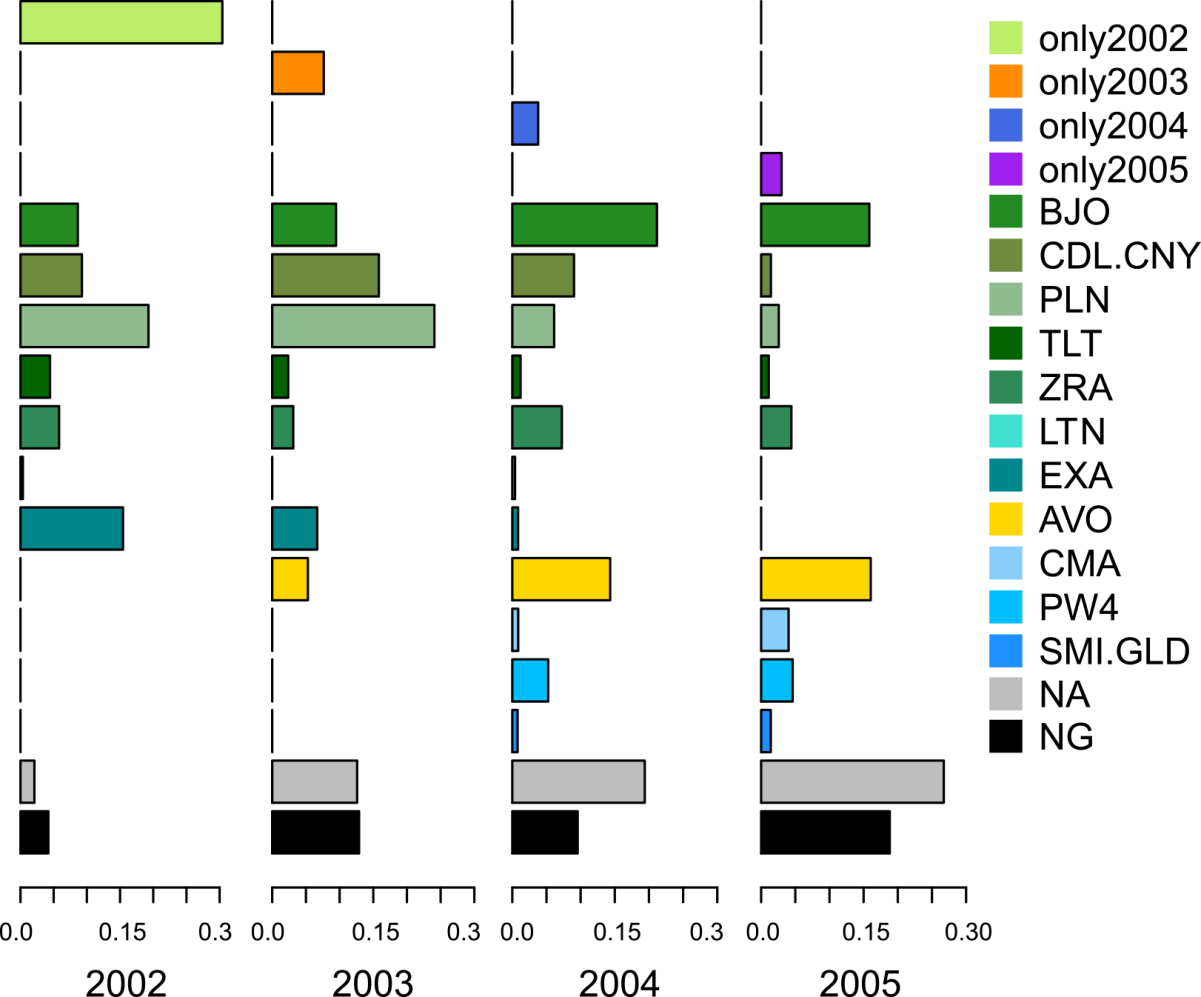
Cultivars assigned to fields from 2002 to 2005, expressed in unit area (hectares). The proportions include fields to which a cultivar could not be assigned (NA, not assigned) and fields not analysed (NG, not genotyped). The x-axis scales are in percentage. Cultivar groupings are defined as follows:—only2002: Cando (CNO), Contact (CNT), Capitol-Carolus (CPL/CRS), Gaspard (GPD), Madrigal (MDL), Navajo (NJO), Orkan (OKN) and Pronto (PRO);—only2003: Zenith (ZNH), Montego (MTG), Mohican (MHN) and Hektor (HKR);—only2004: Express (EXS), Elvis (EVS), Elite (ELE) and Cocktail (CKL);—only2005: VAR003 (V003) and VAR002 (V002).

The 3893 genotypes obtained discriminated 2292 plants (76.15% of field plants were successfully assigned to a cultivar). From 11 to 15 different cultivars were present in each year (Fig 2). We grouped together cultivars that were sown only in one year (only2002, only2003, only2004, and only2005). Five cultivars were common to the four years: Banjo (BJO), Cadillac/Canary (CDL.CNY), Pollen (PLN), Talent (TLT) and Zeruca (ZRA) (Fig 2). These five cultivars constituted a large proportion of the production of OSR, regardless of the year considered. Cultivar Extra (EXA), which was mostly cultivated in 2002, was progressively left over. Cultivar Aviso (AVO) appeared in 2003 in the study area and then became more and more abundant. Cultivars from 2004 such as Campala (CMA) and Pr45w04 (PW4) also seemed to become more widely established. A significant number of fields could not be assigned to one or two cultivars: either the plants in these fields were assigned, but too many cultivars were present overall, or the plants in these fields could not be assigned. The proportion of fields not assigned to a particular cultivar increased from 2002 to 2005 (10.48% in 2002, 13.33% in 2003, 17.20% in 2004 and 36.05% in 2005).

**Fig 3:**
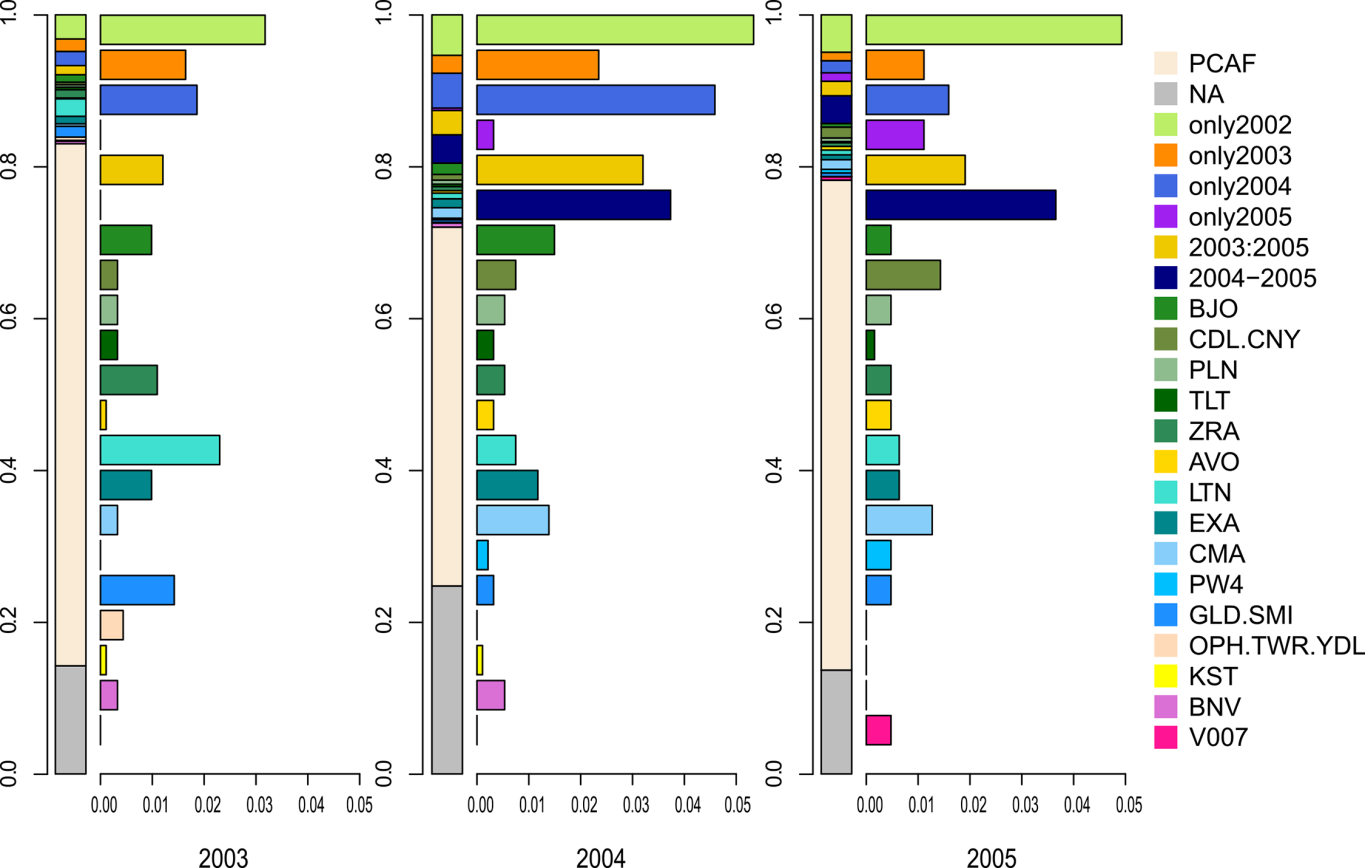
Cultivars assigned to plants from fields from 2003 to 2005 (only assigned fields are represented). For the fields for which a cultivar was assigned, we represent the genetic information at the plant scale. The plants assigned to a cultivar that matches the cultivar of the whole field to which the plant belongs are grouped into a category named PCAF (for Plant whom Cultivar is Assigned to the Field). For the plants for which the cultivar differed from the cultivar assigned to the whole field to which the plants belonged (not PCAF), we used the same cultivar groups as in Fig 2, plus two supplementary groups established for cultivars associations persisting multiple years: - 2003:2005 (i.e. from 2003 to 2005): Aligator (ALR), Bristol (BRL), Columbus (CLS), Expert (EXP), Synergy (SNY) and Savannah (SVN); - 2004–2005: Cap-vert (CPV), Eurol (ERL), VAR004 (V004), VAR005 (V005), VAR001 (V001), Star (STR), Maxol (MXL) and VAR006 (V006). For each year, the first vertical barplot corresponds to the cultivar assignment of all the field plants, according to these groupings. The y-axis scale is expressed in proportion of plants (i.e. 0.2 means 20%). Aside, the horizontal succession of barplots is a zoom of the “not PCAF” plants of this vertical barplot. The x-axis scales are expressed in proportion, from 0 to 0.05 (i.e. 0% to 5%).”

The results differed between the cultivars assigned to the plants in respective fields (plant scale) and the cultivars assigned to the fields themselves (field scale) (Fig 3).We used groupings of cultivars established at the field scale. We grouped together plants that shared their cultivar type with the field to which they belonged. This representation enabled us to highlight plants that were generally considered to have arisen from seed lot contamination, and not from other contamination sources. In terms of the results for each year, plants in fields were assigned to 34 different cultivars in 2003 and 42 different cultivars in 2004 and 2005. When only taking into account plants in assigned fields, the numbers of cultivars were 32 in 2003, 39 in 2004 and 38 in 2005. Cultivars that had been sown in only one year were common in fields, regardless of the year considered. Some cultivars were also detected the year before they were supposedly sown, i.e. cultivars supposedly planted in 2004 were found in 2003, and those believed to be planted in 2005 were found in 2004. Six cultivars that were not assigned at the field scale were assigned at the plant scale: Olphi (OPH), Tower (TWR), Yudal (YDL), Kosto (KST), Bienvenu (BNV) and VAR007 (V007). Plants assigned to the same cultivar as the whole field to which they belonged only represented from 47.3% to 68.8% of the total plants in fields in each year. In contrast, plants assigned to a cultivar different from the cultivar assigned to the whole field to which they belonged ranged from 17% to 28% in each year.

#### Feral plants

**Fig 4:**
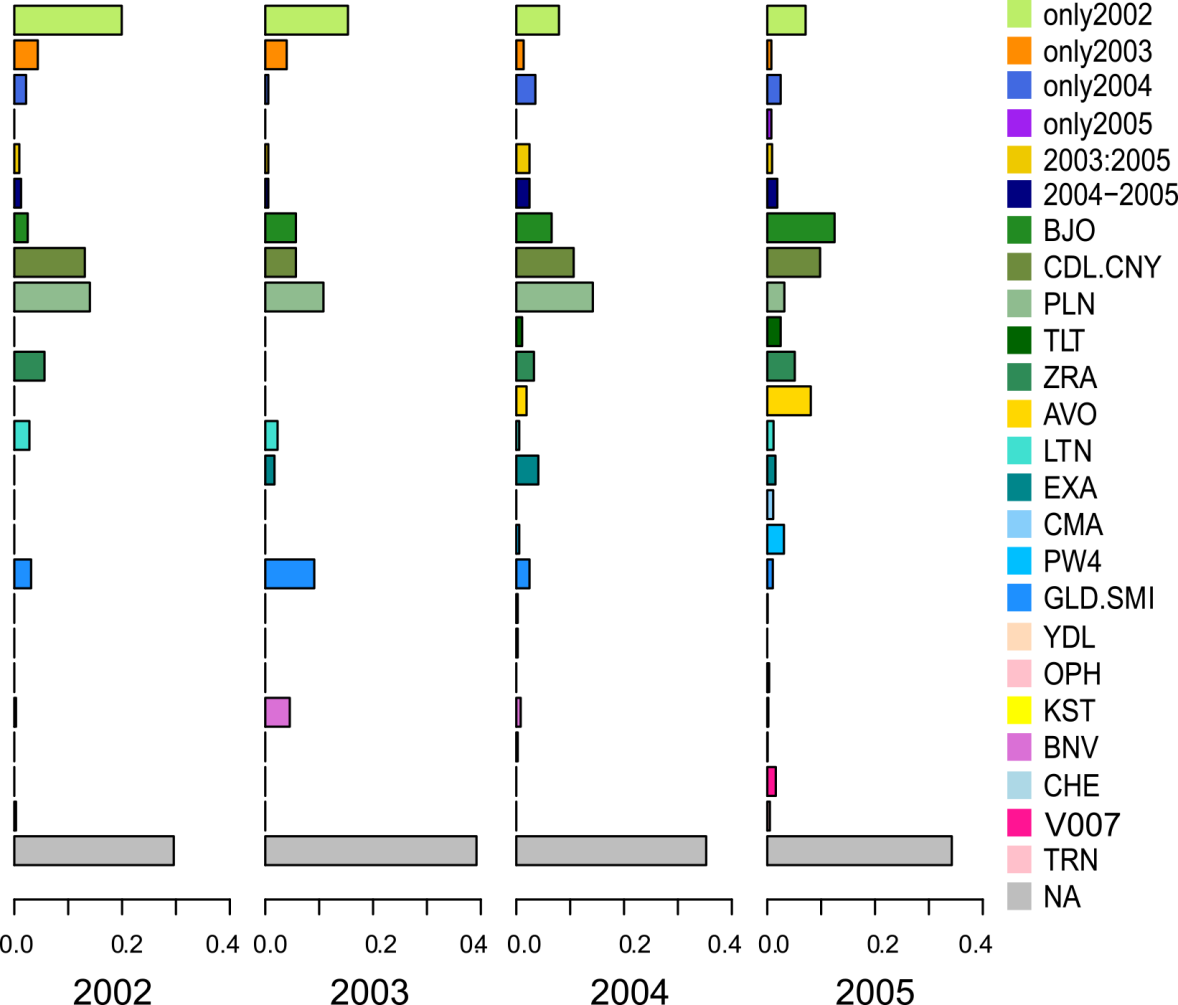
Cultivars assigned to plants from feral populations from 2002 to 2005, in terms of the number of plants assigned. The x-axis scales are in percentage. The cultivar groupings are the same as those in Fig 3.

A total of 6080 genotypes allowed to assign 1521 plants (i.e. 66.13% of feral population plants were assigned to a cultivar). The number of cultivars generally increased with time: 19 different cultivars were identified in 2002, 16 in 2003, 33 in 2004 and 44 in 2005. The proportion of plants that could not be assigned to a cultivar was larger for feral plants (Fig 4) than for field plants (Fig 3). The five cultivars that were present in fields every year (BJO, CDL.CNY, PLN, TLT and ZRA) were also common in feral populations. One “new” cultivar (i.e. not present among the cultivars found in fields and field plants) was found among the feral populations: Cheyenne (CHE), in 2004 and 2005. Cultivars that were sown only in one year in fields were found in feral population over several years. Their proportions decreased over time (Fig 4), unless these cultivars were present before their official selling date [4].

### Exclusion probability

The original exclusion probability was calculated to be 94.98%. This exclusion power indicated that some cultivar were compatible with more than one genotype. The exclusion probability derived from the cultivar frequencies was evaluated to be 90.4%. This difference signifies that plants belonging to cultivars that were discriminated less than expected were common in our dataset. The derived exclusion probability was also moderated because several cultivars shared some genotypes. Therefore, this probability was similar to the exclusion probability previously calculated for only 17 cultivars in the same area [43] instead of our 58 cultivars.

### Cultivar diversity of interfield and interferal populations

**Fig 5:**
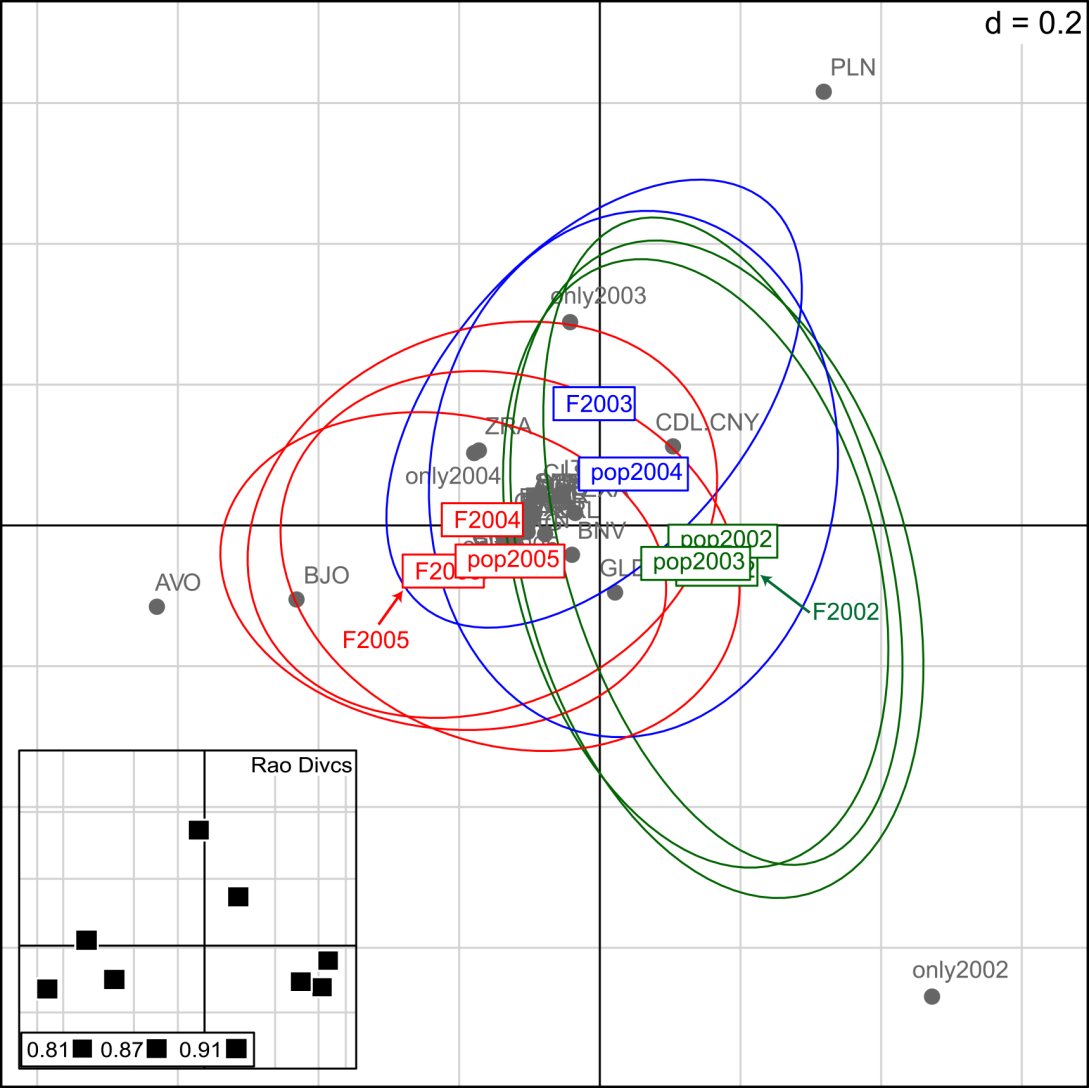
DPCoA of cultivar composition of plants from field and feral populations from 2002 to 2005. DPCoA: Double Principal Coordinate Analysis. Groupings between field and feral populations (in colour) were based on the Rao dissimilarity index calculated from their cultivar compositions (in grey): the green group is F2002, pop2002 and pop2003; the blue group is F2003 and pop2004; and the red group is F2004, F2005 and pop2005. The further the cultivar is from the centre, the more it contributes to the group’s constitution. *Rao Divcs* indicates Rao diversity indexes for each field and population: the larger the square is, the bigger the diversity is. Group pop2002-F2002-pop2003 was characterised by cultivars only sown in fields in 2002 (only2002). Group F2003-pop2004 was characterised by 2003 field cultivars, and the abundance of cultivars Pollen (PLN) and Cadillac/Canary (CDL.CNY). Lastly, group F2004-pop2005-F2005 was characterised by the abundance of Aviso (AVO) and Banjo (BJO) cultivars.

We observed strong similarity among: (i) 2002 feral populations (pop2002 in Fig 5), 2002 field populations (F2002) and 2003 feral populations, (ii) 2003 field populations and 2004 feral populations, and (iii) 2004 field populations, 2005 feral populations and 2005 field populations. The cultivar compositions and cultivar abundances of fields a particular year were similar to those of feral populations in the following year. Cultivars and cultivar abundances were characteristic of those groups, i.e. i, ii and iii, allowing them to be distinguished. Each group was characterised by high internal diversity, regardless of the group (field or feral population).

### Focus on feral population diversity

Feral cultivar diversity was analysed according to the type of roads where feral populations were located (on path verges or paved road verges). This study on feral populations in 2005 was performed because the highest number of feral populations were sampled in that year and because the populations were large enough to study diversity. A total of 91 feral populations with more than four assigned plants were sampled in 2005: 45 on path verges and 46 on road verges.

**Fig 6:**
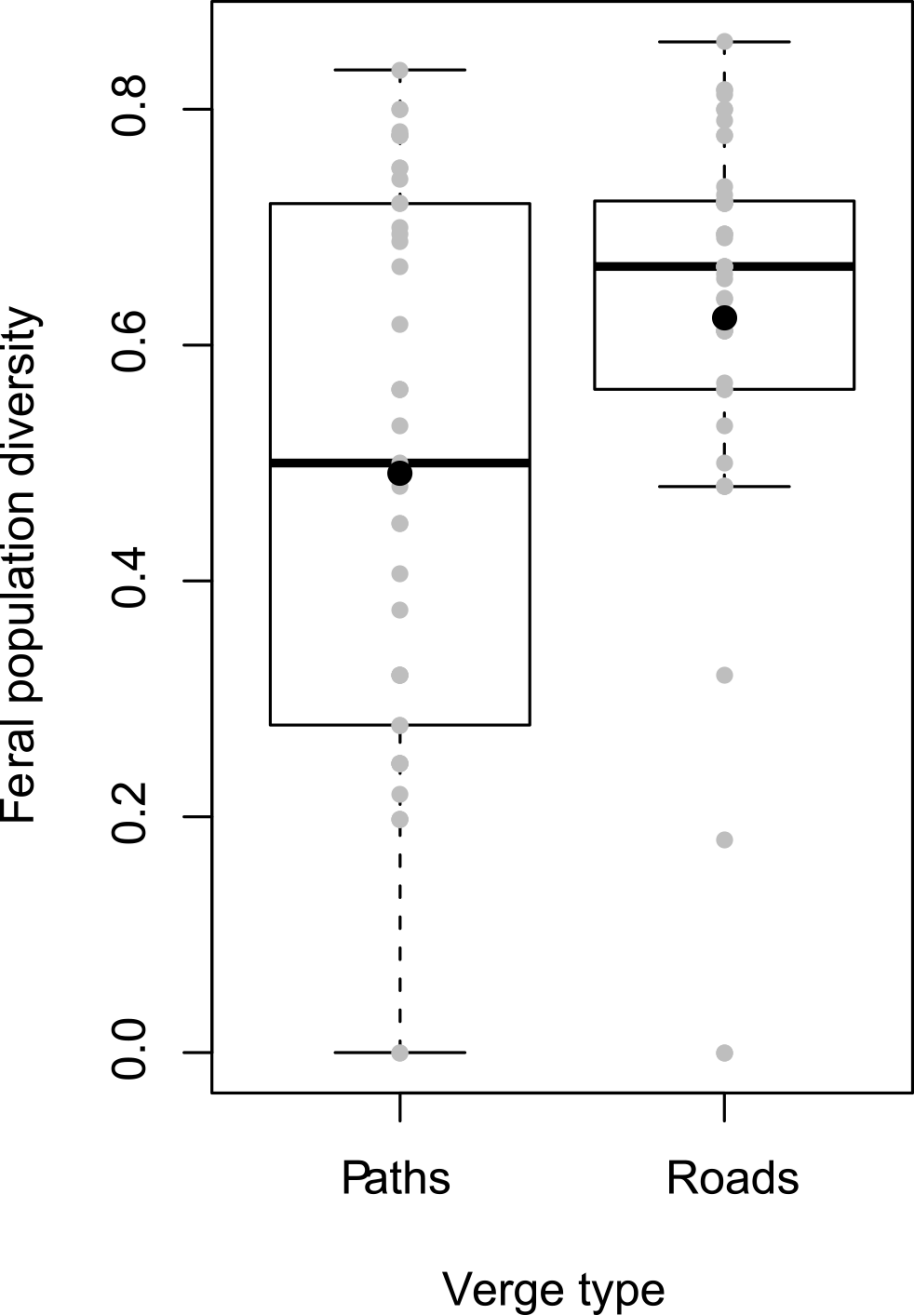
Internal diversity of feral populations in 2005 depending on their localisation. Grey dots indicate diversity indices for each population. Black bars indicate medians. Black dots indicate means.

The mean diversity of feral populations on road verges was greater than that on path verges (Wilcoxon test, p-value = 0.018). Feral populations on path verges were thus more homogeneous in terms of cultivars than feral populations on road verges (Fig 6).

## Discussion

In this study, we highlight variations in the genetic diversity of OSR in an agroecosystem. We have identified three types of field: fields sown with a single cultivar, fields sown with two cultivars and fields unassigned with regard to cultivars. The distribution of cultivars in fields reflected the conventional view of agroecosystems quite well: that is, there was a succession of cultivars, some grown for longer than others because of good performance, some used for one year and then abandoned, and others gradually adopted. Contrary to a certain view [49,50], fields of OSR are not uniform entities in terms of the genotype or even the cultivar.

The existence of farm-saved seeds could explain the genetic composition of the unassigned fields. Although this practice is increasingly discouraged, we know that 15%–30% of farmers (depending on the year considered) kept some of the harvest for sowing the next year [51]. Plants from farm-saved seeds had genotypes that were not in our list of cultivars and thus were not assigned. Farm-saved seeds could partially explain the diversity of cultivars observed. Certified seed lots can also be problematic, for example, OSR certified seed lots are known to potentially be contaminated: by volunteers in certified fields [11,38], by cross-pollination with nearby fields [37], or during the cleaning of mowers and seed trucks [52]. Recently, wheat seed lots have been suspected to be contaminated with GM OSR seeds [20]. Thus, contamination could originate from adjacent fields as well.

The number of cultivars sown each year in this agroecosystem was relatively constant. However, the diversity of cultivars within and between fields was higher than expected (Fig 3). This diversity of cultivars was not related to the protocol of sampling them at the edges of fields. Indeed, we did not find any difference in cultivar diversity between the edges of fields and the middle of fields (S1 Supporting Information). The high level of cultivar diversity may be due to a combination of factors: farm-saved seeds, the contamination of seed lots and the presence of seed banks in fields. Cultivars actively grown in only one year persisted in the agroecosystem in the following years, as field plants (Fig 3) and feral plants (Fig 4). Some cultivars were also detected the year before being identified in fields. However, not all fields in our area were analysed (Table 1). Thus, either these cultivars may have been sown in those fields the year before their appearance or in fields outside of our study area and spilled during transport to Selommes silo, either these cultivars were presents as seed lot contamination in fields previously sown.

As expected, the cultivar composition of plants of feral populations was similar to that of field plants. All of the cultivars, even those that were not dominant in fields, were also found in plants of feral populations. A single “unexpected” cultivar (CHE, Cheyenne) was present among the plants of feral populations. This new cultivar could have been derived either from a truck transporting the harvest from a field outside of the study area, or from a field in the study area that was not analysed. The persistence of cultivars, observed in the case of field plants, was even more important in the case of plants of feral populations. Indeed, we observed a gradual increase in the number of cultivars found in feral populations with time. This increase in identified cultivars might be related to the greater effort to sample feral populations in 2005 (Table 1), but the number of observed cultivars increased in 2005 even we only consider a similar number of sampled plants as in the earlier years. In addition, more plants from feral populations were not assigned to a cultivar than those from fields. Since there was no more missing data in terms of the genotypes of field plants than for plants from feral populations (Table 1), these unassigned plants were probably intercultivar hybrids.

We have shown similarity between the cultivars of field plants in a particular year and the cultivars of feral population plants in the following year (Fig 5). The cultivar diversity of feral populations was also largely similar to the cultivar diversity of fields in the previous year. These results, obtained using genetic tools, confirm the results of Pivard et al. [17], who found that, in the same agroecosystem, 35%–40% of the feral populations in one year resulted from seed immigration from neighbouring fields in the previous year. However, it should be noted that, in terms of the cultivar diversity in the fields, the diversity observed in the present work was observed at the level of field plants, not whole fields.

With our present data, it was not possible to perform both genetic and spatial analyses to link a feral plant to a specific field as putative origin. Only a few feral populations had a sufficient number of plants to be able to assign a cultivar type to them. Moreover, feral populations were spatially fragmented in each year. Only a very small proportion of the feral populations that we sampled was found in two consecutive years, which prevented study of the evolution of cultivar diversity of a persistent feral population. In addition, the fact that, for example, a plant from a feral population assigned to cultivar *X* in 2005 was spatially close to a field also assigned to *X* in 2004 does not give us any information that an *X* feral population plant comes from a seed from this *X* field, but simply shows that the two plants share the same cultivar, without an appropriate model explaining why they do this. For example, the maximum likelihood model [53] that infers the age of a population cannot be applied to our data. This model implies that OSR F_1_ hybrids came from single genotypes whereas our cultivars contain several genotypes in general.

We found that the cultivar diversity of feral populations in road verges was higher than the diversity of feral populations in path verges. This gave an important clue about seed flows on verges. We can assume that the seed losses from fields, from seed banks and from the self-recruitment of feral populations were not significantly different between path and road verges. Animals are not involved in OSR dispersal [25]. Therefore, this difference of cultivar diversity among feral populations can only be explained through traffic dispersal. Traffic on paths is low compared with that on paved roads (pers. obs.) and traffic intensity is linked to the amount and origin of seeds spilled [22]. As such, road verges may receive more seeds from multiple and more or less distant fields and, thus, more seeds of multiple cultivars than path verges. This dispersal could certainly provide a number of seeds of different cultivars sufficient to explain the higher cultivar diversity on road verges.

Although previous studies only considered feral populations and cultivars, they all obtained similar findings about the persistence of feral populations and their effects on coexistence between GM and non GM crops [40–42]. Because of high genetic differentiation between feral populations and cultivars, Bond et al. [41] and Pascher et al. [40] concluded that feral populations maintained themselves over time by self-recruitment and hybridization with cultivars in fields. In this study, we were able to assign a cultivar to most of the feral plants. This difference could be explained by the fact that we worked with 58 cultivars, selected via a survey of farmers, and at a larger spatial scale scale than in the earlier studies. We have also shown the persistence of cultivars through feral plants and, furthermore, through field plants, which highlights the link between feral populations and field plants in the previous year. This additional information led us to the conclusion that, in the context of coexistence between GM and non GM crops, feral populations expressing GM traits might not only be the result of the escape of GM material once and its persistence in feral plants, but also the result of GM persistence among field plants and escape towards feral plants each year. Our results confirm the persistence of feral populations. These populations could serve as a relay for GM contamination of fields.

## Synthesis

This study showed that agroecosystems operate in a complex manner, and that human activities have a strong impact on the dynamics of plant species in these agroecosystems. Oilseed rape fields are not uniform entities, that is, products of sowing a single cultivar. Fields have a cultivar footprint depending on the cultivars previously grown in the agroecosystem. Residual cultivars replace each other over time. Feral populations have a level of cultivar diversity similar to that of fields. However, the cultivar diversity of feral populations increases over time as a result of their persistence due to seed losses from fields, survival in seed banks, self-recruitment into feral populations and dispersal by traffic. Using genetic tools, we have demonstrated the existence of a link between the cultivar diversity of feral populations in a particular year and the cultivar diversity of fields in the previous year. This is the first time, to our knowledge, that the link between feral populations and field plants in the previous year has been highlighted by using molecular markers in an agroecosystem. These findings should be incorporated into gene flow models in order to improve assessments of the functioning of agricultural landscapes and thus the impacts of the introduction of GM organisms.

## Supporting Information

S1 Supporting Information Sub-experiment on genotypes diversity in fields and feral populations. (DOCX)

S2 Supporting Information Maximum likelihood assignment method. (PDF)

S3 Supporting Information Exclusion probability. Data accessibility: Feral and cultivars information and genotypes could be found on DRYAD (doi: 10.5061/dryad.pc6sd).
(PDF)

S1 Fig Figures of the S1 Supporting Information. Figure A: Sub-experiment sampling protocol. Details about the sampling protocol for the 5 fields (C355, C151, C704, C583 and C6) and the 3 feral populations (F13, F222 and F86) with transects information. Leaves were taken approximatively every five meters along transects. Transects inside fields ranged from 5 meters to 75 meters from edge. Figure B: Genotypes abundance table for fields. The different genotypes are listed in column and the plants are classified by fields and then by transect. Each black squared indicated a match genotype/plant. Figure C: Genotypes abundance table for feral populations. The different genotypes are listed in column and the plants are classified by population and then by transect. Each black squared indicated a match genotype/plant. Figure D: Samplings with replacement and 1000 replicates of from one leaf to the maximal number of leaves of a field. If present, points indicate true values. Lines represent smoothed distributions. Straight and large dashed lines indicate true mean number of genotypes. Curved and small dashed lines indicate 95% confidence intervals. a: observed frequency of unique genotypes in fields; b, main genotypes frequencies; c, mean number of genotypes in the sub-sample; d, number of different genotypes; e, frequency of the field attribution to the main genotype when 4 leaves at least presents this genotype and f, frequency of the field attribution to the main genotype when 6 leaves at least presents this genotype. Figure E: Samplings with replacement and 1000 replicates of from one leaf to the maximal number of leaves of a feral population. If present, points indicate true values. Lines represent smoothed distributions. Straight and large dashed lines indicate true mean number of genotypes. Curved and small dashed lines indicate 95% confidence intervals. a: observed frequency of unique genotypes in feral populations; b, main genotypes frequencies; c, mean number of genotypes in the sub-sample; d, number of different genotypes; e, frequency of the feral population attribution to the main genotype when 4 leaves at least presents this genotype and f, frequency of the feral population attribution to the main genotype when 6 leaves at least presents this genotype.
(PDF)
