# Supplementary material for: Genetic Diversity of Oilseed Rape Fields and Feral Populations in the Context of Coexistence with GM Crops
Source: PLoS One. 2016 Jun 30;11(6):e0158403. doi: 10.1371/journal.pone.0158403 (PMC4928878; doi:10.1371/journal.pone.0158403)
Supplement: S1 Supporting Information — (DOCX) [file pone.0158403.s001.docx]

S1 Supporting Information: **Sub-experiment on genotypes diversity in fields and feral populations.**

Background

The goal of this sub-experiment was to assess the quality of the experimental protocol. By sampling plants on the border of fields, we could collect more genetic heterogeneity than inside fields. Moreover, sampling 10 plants could not be representative of the genetic diversity of a whole field or feral population. These questions were addressed from an extent sampling design compared to regular sampling design.

**Materials and Methods**

In 2007, a supplementary sampling operation was performed on 5 fields and 3 feral populations. 60 leaves were collected in the fields along transects (one leaf per 5 meters and an average of 15 leaves per transects), and 50 leaves (except F222, 60 leaves) were collected for the feral populations (see S1 Figure A for sampling design).

The leaves were treated with the same methodology present in the current paper. We obtained genotypes from 8 SSR. Using cultivar in the analysis was not possible. We tried to assign our samples, as the dataset contained almost no missing values. Less than half of the genotypes were assigned to a cultivar. In consequence, the biologic unit here is the genotype. We thus examined the distribution of genotypes along transects for the 5 fields and the 3 feral populations.

To assess the statistical significance of our current protocol (10 plants), we realized for each field and for each population genotypes random sub-samplings with replacement among all the available genotypes. We sub-sampled 1000 times from 1 plant to the maximum number of plants for each fields and populations. These samplings (S1 Figures D and E) enabled the computation of the frequency of the dominant genotype (b), the mean number of genotypes in the sampling (c), the number of different genotypes (d), the frequency of assignment to the dominant genotype when at least 4 leaves belonged to this genotype (e) and at least 4 leaves belonged to this genotype (f). We also represented the abundance of each genotype in the total sampling (a).

**Results and discussion**

**Genetic diversity in fields and populations** (S1 Figures B and C)

These representations showed the unique genotypes of individuals, by transect then by field or population. The fields are more homogeneous than the feral populations. There are 56 unique genotypes in the 256 fields’ leaves, against 89 unique genotypes in the 156 feral populations’ leaves. Generally, there is one dominant genotype by field. For 2 fields (C151 and C355), the dominant genotype is the same. This dominant genotype is present in all theirs transects. The other genotypes, often single in abundance, are present on the border or inside the field. Many genotypes seem to cohabit with the dominant genotype.

The transects along the feral populations give similar results. If there are several leaves with the same genotype, this common genotype is not restricted to a single transect. Single genotypes are distributed all along the transects. Apriori, a sub-sampling on this important number of non-dominant genotypes would only result in a heterogeneous group of genotypes, varying at each sampling and transect. The main difference with fields is that there is no dominant genotype as in fields: only few genotypes that are shared by more than one leave.

In summary, there seems to be no sampling bias when sampling plants along the border of the fields rather than inside them. As for feral population, the genetic diversity seems so important that it seems impossible to obtain a representative sub-sampling.

**Effect of the sub-sampling on the fields (S1 Figure D).**

The frequency of the dominant genotype (b) depends from its abundancy in the sample (a) but also from the abundancy of the other genotypes. Thus, the more different genotypes in the sampling, the harder getting a representative sub-sampling (c, d). Even when as many leaves as existing in the fields C704 and C6 were sampled, with replacement, their sub-samples did not represent their complete genotypic diversity.

Thus, if a sampled field contains one main genotype, and if this genotype is as frequent as main genotypes are in our sub-experiment, taking 10 leaves from this field and decide that a minima 4 genotypes should be equal to this main genotype is enough to correctly assign the genotype (and thus, the cultivar) to the fields. For uniform fields, setting 4 identical genotypes seems correct. But with the strict criteria of a minimum 6 genotypes, taking 10 leaves is not enough to obtain a correct frequency (f). A sampling of 20 leaves at least should have been consider, and even more for the fields C704 and C6.

**Effect of the sub-sampling on the feral populations (S1 Figure E)**

The feral populations contain more different genotypes and sometimes up to 2 main genotypes (F222 and F86). The frequency of the dominant genotype (b) is relatively well evaluated, but very low. Evermore, the sub-sampled genetic diversity did not represent the existing genetic diversity (c and d) in any of the samplings. In this case, assigning a feral population to a genotype as fields does not have any meaning with fields criteria (e and f) since there are almost as many genotypes as there are individuals.

The high genotypic diversity is probably the consequence of the possible multiple origins of feral populations. Feral populations may come from dispersal events of one unique cultivar each, and so contains one or two main genotypes. But in the same time, feral plants could origin from reproduction inside the feral population or from seed banks established years ago.

**Conclusions on the experimental protocol**

We had no prior knowledge when we chose the fields for the sub-experiment in 2007. We thus did not know if each field could contain one cultivar, two cultivars or could be sown with farm saved seeds. Each year, more than one hundred of fields are sown in Selommes area and hundreds of feral populations grow on the side of the roads. Thus, it was important to determine a number of plants giving enough genetic information to characterize fields and populations while staying an economically acceptable cost, giving the high number of fields and feral population to survey. Moreover, more than one border was genotyped for some fields while both leaves and seeds could have been genotyped.

Furthermore, the biologic unit here was the genotype, and not the cultivar. The genotypes present in these fields probably belong to the same cultivar, which would increase the probability of assignment of the field to a given cultivar.

For fields, this number of 10 plants, even if less satisfying if the genetic diversity of the field is high, is a good compromise enabling the analysis of a maximum of borders while having reliable genetic information on the main genotype if the field is homogeneous. However, this compromise is not satisfying enough when working on the genetic diversity of the feral populations. The only thing well estimated is the main genotype frequency, and this frequency is well assessed with only 10 leaves. But this main genotype frequency is so low that we cannot assign a cultivar to feral population. As the genetic diversity of our feral population is so high that even 50 leaves would not have been enough to correctly assess it, we assume a much dense sampling design would probably result in more genetic diversity.
