# Supplementary material for: Genetic Diversity of Oilseed Rape Fields and Feral Populations in the Context of Coexistence with GM Crops
Source: PLoS One. 2016 Jun 30;11(6):e0158403. doi: 10.1371/journal.pone.0158403 (PMC4928878; doi:10.1371/journal.pone.0158403)
Supplement: S2 Supporting Information — (PDF) [file pone.0158403.s002.pdf]

S2 Supporting Information: Maximum likelihood assignment method.

The likelihood that seed genotype *Gi* belongs to a plant of cultivar *v* was calculated as:

$$L(G\mathrm{i}, v) = \sum_{gv} f\mathrm{gv} \cdot [sT(G\mathrm{i}/M\mathrm{gv}, M\mathrm{gv}) + (1 - s) \cdot \sum_{gv\prime} f\mathrm{gv}'T\ (G\mathrm{i}/M\mathrm{gv}, F\mathrm{gv}'),$$

where *gv* represents all of the possible genotypes for the mother and *gv'* for the father, both of cultivar *v*; $f\mathrm{gv}$ and $f\mathrm{gv}'$ are the genotype frequencies of *gv* and *gv'*; and *s* is the selfing rate [s = 0.63, 17]. $T(G\mathrm{i}/M\mathrm{gv}, F\mathrm{gv}')$ is the Mendelian segregation probability that a mother of genotype *gv* and a father of genotype *gv'* produce offspring of genotype *Gi*.
