# Supplementary material for: Genetic Diversity of Oilseed Rape Fields and Feral Populations in the Context of Coexistence with GM Crops
Source: PLoS One. 2016 Jun 30;11(6):e0158403. doi: 10.1371/journal.pone.0158403 (PMC4928878; doi:10.1371/journal.pone.0158403)
Supplement: S3 Supporting Information — Data accessibility: Feral and cultivars information and genotypes could be found on DRYAD (doi: 10.5061/dryad.pc6sd). (PDF) [file pone.0158403.s003.pdf]

**S3 Supporting Information: Exclusion probability.**

We adapted the exclusion probability of Devaux et al. [43] derived from a previous paper [47] to take into account cultivar richness:

$$P = \sum_{v'} N\mathrm{v}' \sum_{v} N\mathrm{v} \sum_{k} g\mathrm{v}, \mathrm{Gk} \cdot \frac{1}{2\mathrm{n}} \sum_{i} 1\mathrm{incompatibility} \cdot (\gamma_{\mathrm{v}',\mathrm{Gk},\mathrm{i}}, v'),$$

where $N\mathrm{v}$ is the OSR plant number assigned to a cultivar *v* in a given year; n is the number of loci; $g\mathrm{v}, \mathrm{Gk}$ is the frequency of genotype $G_k$ of cultivar *v*; and $\gamma\mathrm{v}', \mathrm{Gk}, \mathrm{i}$ represents the rank *i* of the gamete produced by the genotype $G_k$ of cultivar *v*. $\sum_i 1\mathrm{incompatibility} \cdot (\gamma\mathrm{v}', \mathrm{Gk}, \mathrm{i}, v')$ is an incompatibility function between gamete g and cultivar *v*. This function returns 1 if g and *v* are incompatible, but 0 if they are compatible.
