# Supplementary figures and images for: Genetic Diversity of Oilseed Rape Fields and Feral Populations in the Context of Coexistence with GM Crops
Source: PLoS One. 2016 Jun 30;11(6):e0158403. doi: 10.1371/journal.pone.0158403 (PMC4928878; doi:10.1371/journal.pone.0158403)

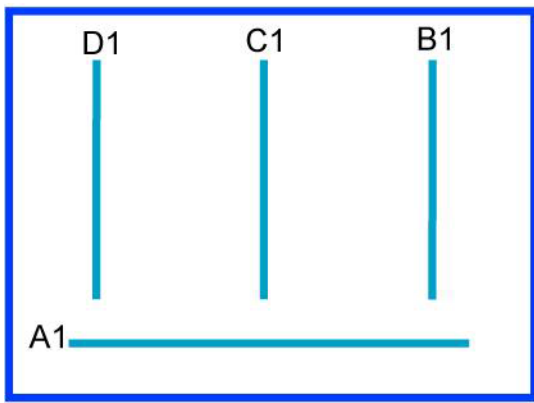

C355

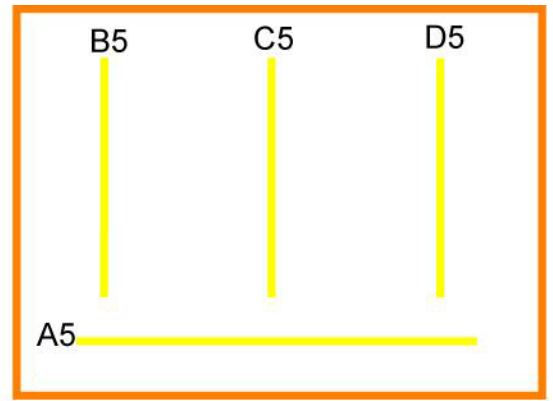

C151

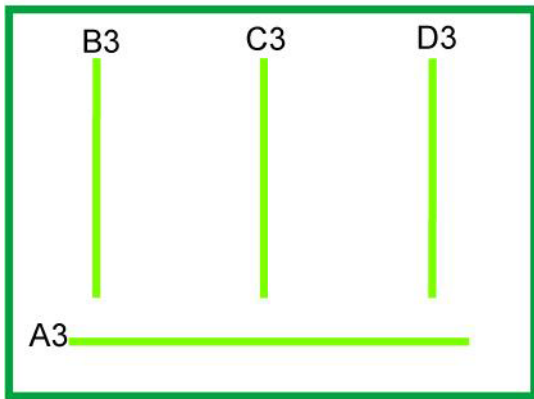

C704

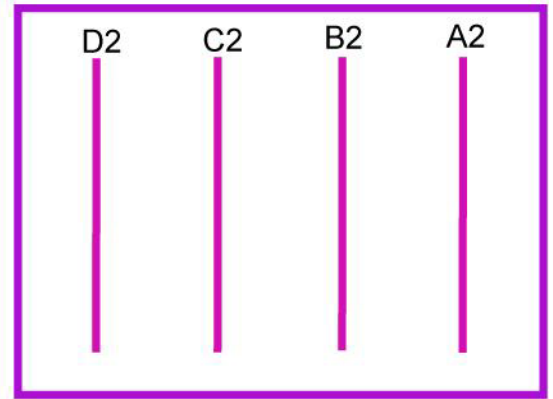

C583

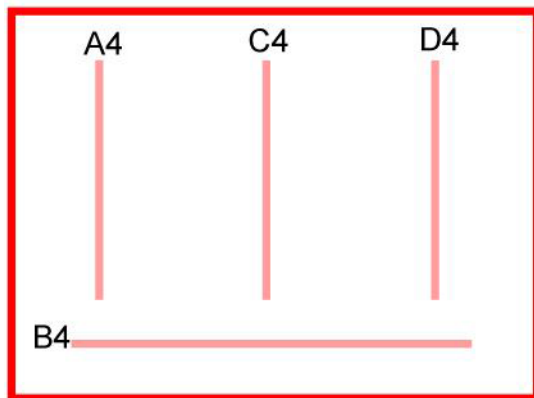

C6

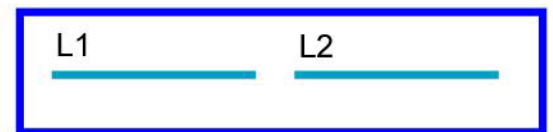

F13

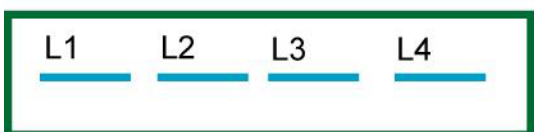

F222

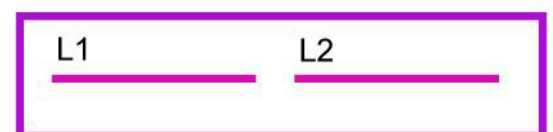

F86

Figure A.

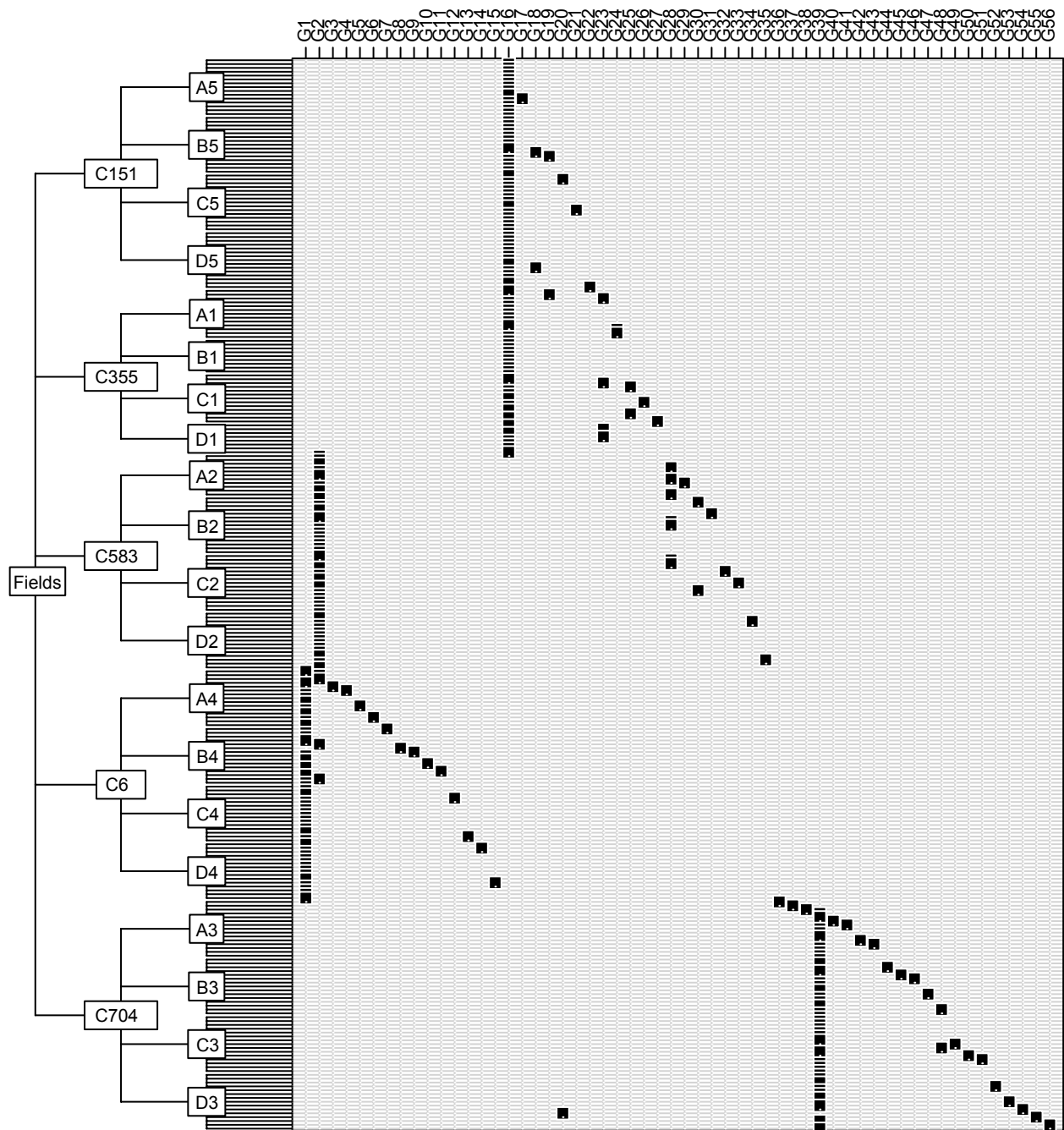

Figure B.

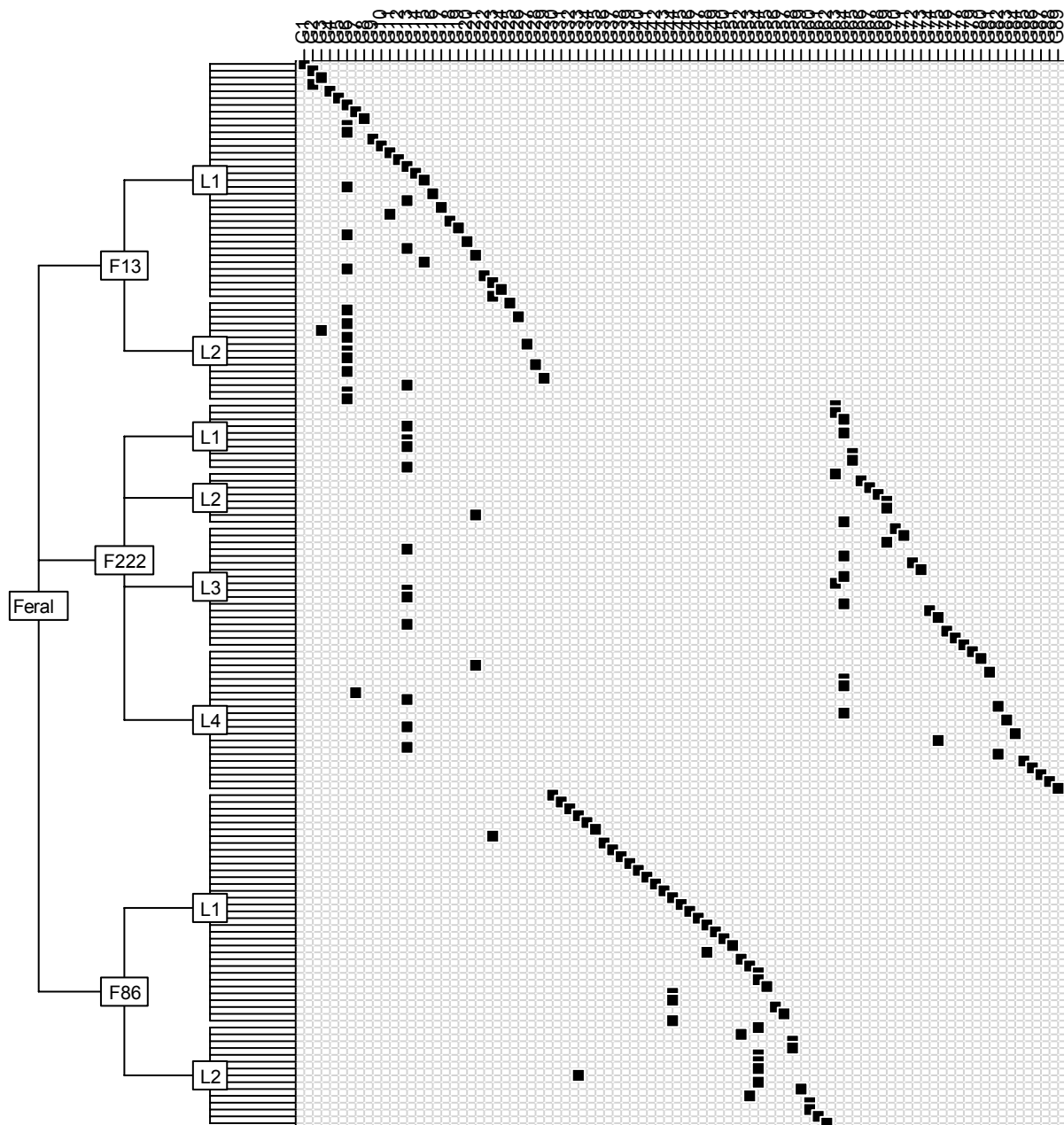

Figure C.

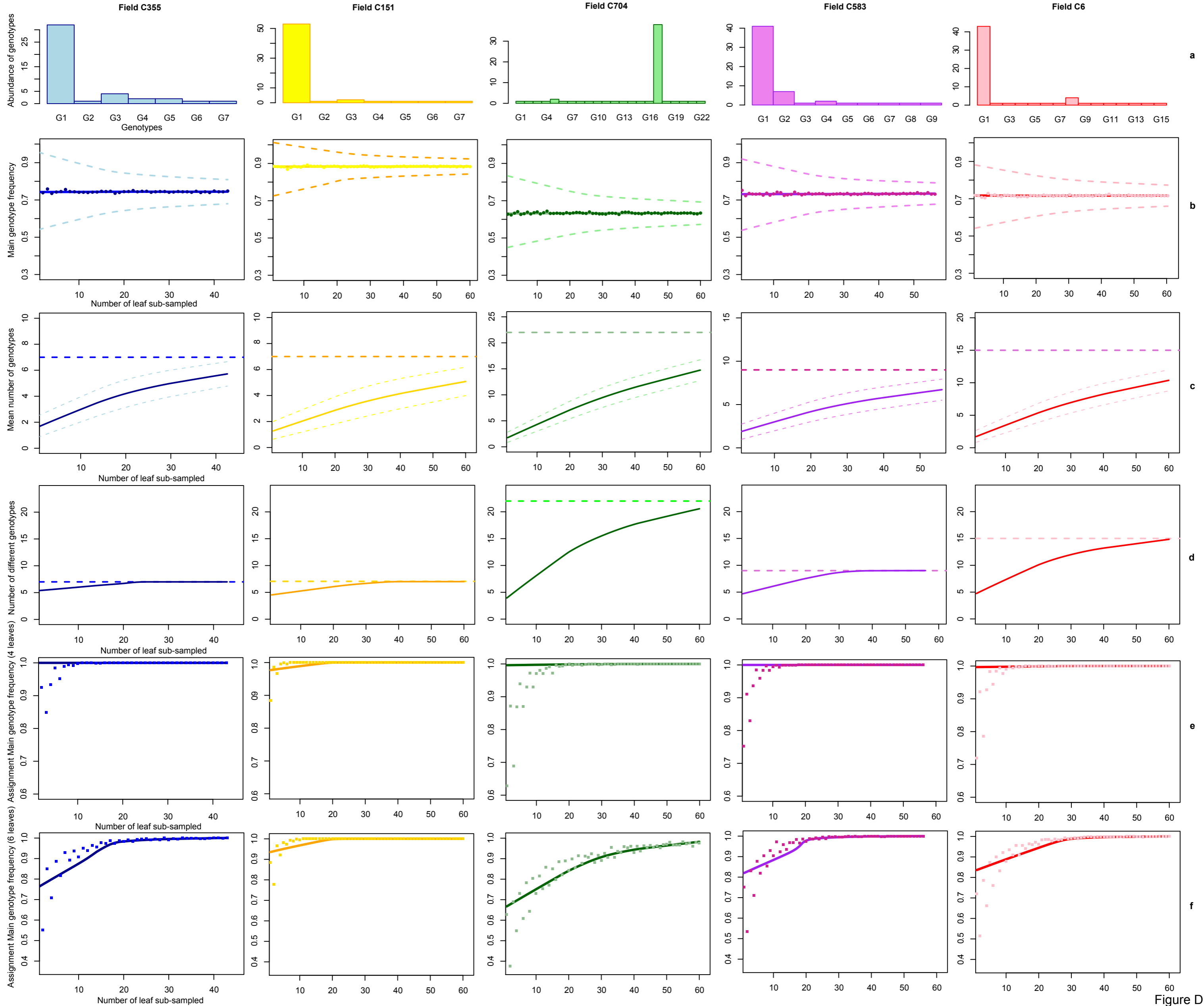

Figure D

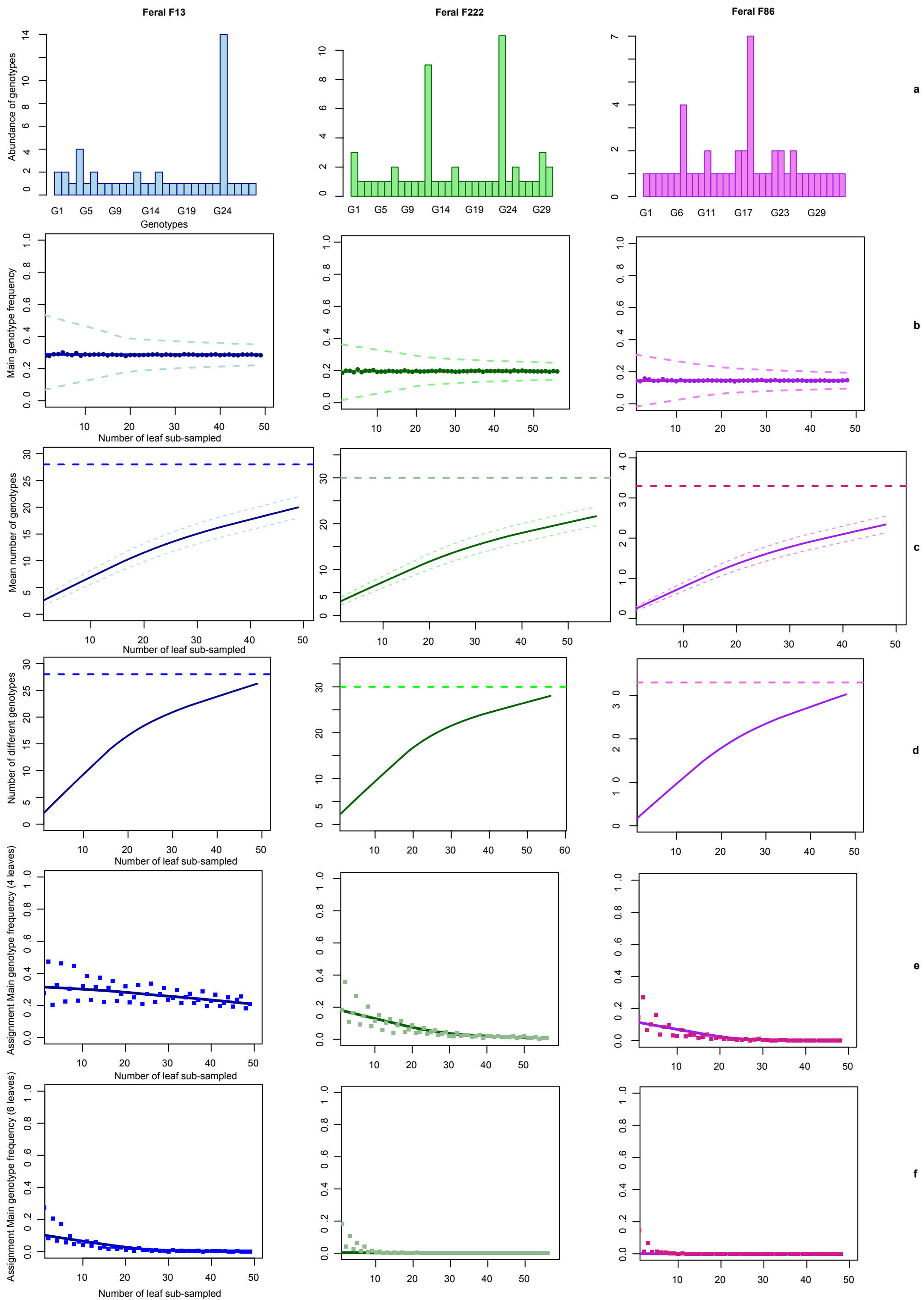

Figure E.

Supplement: S1 Fig — Figure A: Sub-experiment sampling protocol. Details about the sampling protocol for the 5 fields (C355, C151, C704, C583 and C6) and the 3 feral populations (F13, F222 and F86) with transects information. Leaves were taken approximatively every five meters along transects. Transects inside fields ranged from 5 meters to 75 meters from edge. Figure B: Genotypes abundance table for fields. The different genotypes are listed in column and the plants are classified by fields and then by transect. Each black squared indicated a match genotype/plant. Figure C: Genotypes abundance table for feral populations. The different genotypes are listed in column and the plants are classified by population and then by transect. Each black squared indicated a match genotype/plant. Figure D: Samplings with replacement and 1000 replicates of from one leaf to the maximal number of leaves of a field. If present, points indicate true values. Lines represent smoothed distributions. Straight and large dashed lines indicate true mean number of genotypes. Curved and small dashed lines indicate 95% confidence intervals. a: observed frequency of unique genotypes in fields; b, main genotypes frequencies; c, mean number of genotypes in the sub-sample; d, number of different genotypes; e, frequency of the field attribution to the main genotype when 4 leaves at least presents this genotype and f, frequency of the field attribution to the main genotype when 6 leaves at least presents this genotype. Figure E: Samplings with replacement and 1000 replicates of from one leaf to the maximal number of leaves of a feral population. If present, points indicate true values. Lines represent smoothed distributions. Straight and large dashed lines indicate true mean number of genotypes. Curved and small dashed lines indicate 95% confidence intervals. a: observed frequency of unique genotypes in feral populations; b, main genotypes frequencies; c, mean number of genotypes in the sub-sample; d, number of differen [file pone.0158403.s004.pdf]
